# A novel multi-model estimation of phosphorus in coal and its ash using FTIR spectroscopy

**DOI:** 10.1038/s41598-024-63672-x

**Published:** 2024-06-14

**Authors:** Arya Vinod, Anup Krishna Prasad, Sameeksha Mishra, Bitan Purkait, Shailayee Mukherjee, Anubhav Shukla, Nirasindhu Desinayak, Bhabesh Chandra Sarkar, Atul Kumar Varma

**Affiliations:** 1 Photogeology and Image Processing Laboratory, Department of Applied Geology, Indian Institute of Technology (Indian School of Mines), Dhanbad, Jharkhand 826004 India; 2 Coal Geology and Organic Petrology Laboratory, Department of Applied Geology, Indian Institute of Technology (Indian School of Mines), Dhanbad, Jharkhand 826004 India; 3 Department of Geology, Ravenshaw University, Cuttack, Odisha 753003 India; 4 Geocomputational and GIS Laboratory, Department of Applied Geology, Indian Institute of Technology (Indian School of Mines), Dhanbad, Jharkhand 826004 India

**Keywords:** Phosphorus, Minor element, Coal, Coal ash, FTIR, Spectroscopy, Environmental sciences, Environmental impact

## Abstract

The level of phosphorus must be carefully monitored for proper and effective utilization of coal and coal ash. The phosphorus content needs to be assessed to optimize combustion efficiency and maintenance costs of power plants, ensure quality, and minimize the environmental impact of coal and coal ash. The detection of low levels of phosphorus in coal and coal ash is a significant challenge due to its complex chemical composition and low concentration levels. Effective monitoring requires accurate and sensitive equipment for the detection of phosphorus in coal and coal ash. X-ray fluorescence (XRF) is a commonly used analytical technique for the determination of phosphorus content in coal and coal ash samples but proves challenging due to their comparatively weak fluorescence intensity. Fourier Transform Infrared spectroscopy (FTIR) emerges as a promising alternative that is simple, rapid, and cost-effective. However, research in this area has been limited. Until now, only a limited number of research studies have outlined the estimation of major elements in coal, predominantly relying on FTIR spectroscopy. In this article, we explore the potential of FTIR spectroscopy combined with machine learning models (piecewise linear regression—PLR, partial least square regression—PLSR, random forest—RF, and support vector regression—SVR) for quantifying the phosphorus content in coal and coal ash. For model development, the methodology employs the mid-infrared absorption peak intensity levels of phosphorus-specific functional groups and anionic groups of phosphate minerals at various working concentration ranges of coal and coal ash. This paper proposes a multi-model estimation (using PLR, PLSR, and RF) approach based on FTIR spectral data to detect and rapidly estimate low levels of phosphorus in coal and its ash (R$^2$ of 0.836, RMSE of 0.735 ppm, RMSE (%) of 34.801, MBE of − 0.077 ppm, MBE (%) of 5.499, and MAE of 0.528 ppm in coal samples and R$^2$ of 0.803, RMSE of 0.676 ppm, RMSE (%) of 38.050, MBE of − 0.118 ppm, MBE (%) of 4.501, and MAE of 0.474 ppm in coal ash samples). Our findings suggest that FTIR combined with the multi-model approach combining PLR, PLSR, and RF regression models is a reliable tool for rapid and near-real-time measurement of phosphorus in coal and coal ash and can be suitably modified to model phosphorus content in other natural samples such as soil, shale, etc.

## Introduction

Coal is one of the most abundant energy sources worldwide, providing reasonable, reliable, and constant power available on demand to meet energy consumption and industrial needs. Coal originates from diverse plants, accumulation of skeletal fragments, and organic elements within peat deposits^1,2^. Over time, coal undergoes extended geological and microbiological processes, incorporating multi-elemental impurities^1^. These multi-elemental impurities in coal greatly influence the environmental, economic, and combustion liability^3^. The elemental composition of coal is an important factor governing the liability of coal to spontaneous combustion, which is one of the most dangerous mining hazards^4–6^. They also affect the quality and pose deleterious effects during coal utilization. In recent years, there has been a growing focus on the determination of elements in coal and coal ash due to their significant role in the local and global energy mix. Phosphorus found in coal plays a crucial role in metallurgical processing, as it significantly impacts the quality of the resulting products. The lack of commercially viable alternative energy technologies that can significantly replace fossil fuels makes coal vital to meeting the global energy demand. Coal generates approximately 40% of the world’s power and heat, despite growing decarbonization efforts over the past 40 years^7^. Hence, there is a critical demand for the advancement of efficient, economical, and swift techniques to determine the elemental content in coal and coal ash samples. Analyzing the elemental impurities within coal will offer direction for refining coal purification methods, thereby enhancing the coal quality. Furthermore, improved knowledge and quantification of the elemental impurities in coal and coal ash will play an important role in their effective utilization, as the elemental makeup of the input coal significantly impacts the final quality of the products and residues.

### Significance of phosphorus content in coal and coal ash

Phosphorus (P), with atomic number 15, is a crucial element for living cells, which, during organic decay in the peat bed, is released from the plant structure and re-precipitated elsewhere. Phosphorus-bearing mineral groups such as apatite, monazite, xenotime, and crandallite are ubiquitous in coal^8,9^. Although most of the phosphorus in coal is thought to be in the form of inorganic mineral components, researchers have identified the possibility of organic phosphorus in coal^9^. The Clarke value of phosphorus is 200 ± 30 ppm for brown coal and 250 ± 10 ppm for hard coal^10–14^. The amount of P in coal varies depending on the location and geological period. Coal from the Cretaceous era found in Western Canada and coal from the Permian era found in India and Australia have higher levels of P compared to coal from the Carboniferous era found in the United States and Europe^15^.

Though present in minor amounts, the presence of phosphorus in coal has garnered considerable attention due to its detrimental effect on the marketing and use of coking coals. Phosphorus is crucial for enhancing the strength of the metal, but excess can render the final metal product brittle^16^. The presence of phosphorus in the form of an oxide impurity results in the formation of intergranular segregation during the process of continuous casting. This results in a decrease in the ductility and toughness of the steel, which, in turn, results in an increased risk of breakage during processing, fabrication, and use^17^. In addition to this, phosphorus can cause deposits to form in the superheater of boilers and can harm catalysts in the process of liquefaction^11^. This reduces the heat transfer rate, increases pressure drop, reduces the overall combustion efficiency, and increases the maintenance cost of the system. Thus, phosphorus impurities in coal will uncover their grade and value. Phosphorus is also significant in the long-term leaching of coal waste products^2,18,19^. Hence, it is important to monitor and control the quality of coal during various processing stages, such as washing, blending, and pulverization, to estimate the likely interactions they may have in different coal utilization processes. This ensures that coal meets all regulatory specifications and can help optimize the performance of coal-fired power plants and other industrial processes that depend on coal as a fuel source. Accurate quantification of phosphorus content can help power plant operators adjust combustion parameters to minimize these effects. Hence, the rapid determination of phosphorus in coal is of primary interest, especially for low phosphorus steel or ferro alloy-making processes where the input material, like coke, may contribute up to 36% compared to 21% from iron ore and 43% of phosphorus from sinter^20–22^.

Massive amounts of coal ash produced from coal combustion can release airborne phosphorus compounds, contributing to PM10 particulate matter. Concerns over long-term phosphorus supply in nature and its environmental impact have arisen. Coal fly ash, rich in essential nutrients, including phosphorus, holds the potential for enhancing plant growth and amending acidic soils. Analyzing the elemental composition of coal ash is crucial due to its dual role as a health hazard and industrial resource^23–27^. Hence, there is a vital requirement for a fast and dependable method to measure and monitor phosphorus levels in coal and coal ash, necessitating the use of cost-effective and expeditious multi-element analytical techniques.

### Phosphorus detection methods in coal and coal ash

Numerous analytical techniques are available for the elemental determination of coal and coal ash. Techniques like traditional wet analysis methods (gravimetric and volumetric) for measuring coal phosphorus are labour-intensive and destructive. While other advanced techniques like inductively coupled plasma optical emission spectroscopy (ICP-OES), inductively coupled plasma mass spectrometry (ICP-MS), Atomic fluorescence spectrometry (AFS), and atomic absorption spectroscopy (AAS) offer efficiency, they involve complex wet digestion sample preparation and high operational costs. Sample preparation requires the use of inorganic acids (HClO_4_, HF, H_2_SO_4_, H_3_BO_3_), which can pose safety risks and negatively impact spectrometric techniques due to matrix effects and spectral interferences^28,29^. Moreover, wet digestion is prone to systematic errors, including contamination and analyte losses, exacerbated by operating at low temperatures and atmospheric pressure, leading to poor elemental extraction recoveries^30^. High carbon concentrations in coal can also cause spectral and non-spectral interferences in inductively coupled plasma-based detection techniques. Excessive acid and residual carbon content alter solution properties, affecting the aerosol formation and analyte transportation to the plasma, resulting in signal reduction and high background, affecting detection limits^31,32^. AFS is vulnerable to specific elements and is impacted by fluorescence and matrix effects, and it may not completely mitigate spectral interference^33^.

Non-dispersion detection methods like X-ray diffraction (XRD), X-ray Fluorescence (XRF), and X-ray photoelectron spectroscopy (XPS) are non-destructive alternatives, with XRF being common for phosphorus measurement and requiring minimal sample preparation. This is advantageous, especially for coal samples, which are difficult to dissolve due to their complex organic matrices. Above all, direct analysis with non-destructive techniques is particularly useful over destructive methods for determining volatile elements^32^. Matrix interferences resulting from major elements comprising the majority of the sample matrix and the need for careful standard utilization reduce the sensitivity for minor and trace elements, leading to high detection limits and poor precision in quantitative analysis. Fractionation and spectrometric interference issues can also significantly disrupt the accuracy and precision of this analysis approach^32,34,35^. Overall, these methods have trade-offs in terms of cost, sensitivity, and practicality for handling bulk sample numbers. For the rapid assessment of phosphorus in coal and its ash, there is a strong preference for an approach that reduces sample processing time and analytical duration, facilitates high sample throughput, and ensures acceptable accuracy.

Until now, a limited number of research studies have outlined the estimation of elements like sulfur and carbon, predominantly relying on FTIR spectroscopy and machine learning techniques^36–43^. Quantifying phosphorus in coal and coal ash using FTIR spectroscopy is challenging due to the element’s extremely low concentration and weak signal. Mid-infrared (MIR) spectra of coal and coal ash contain absorbance peaks due to the functional and anionic groups of minerals related to phosphorus in coal and coal ash. The objective of this current study was to assess the effectiveness of using FTIR spectroscopy in conjunction with multivariate regression techniques for the measurement of phosphorus in coal and its ash, compared to traditional analytical methods. Samples prepared in different concentration ranges, making a total of 96 FTIR spectra each for coal and its ash, were utilized to establish the model. The standard XRF method determined the reference phosphorus concentration in each sample. After establishing a model, it becomes feasible to predict the concentrations of phosphorus in an unknown sample by analyzing its spectrum in near-real time.

**Figure 1:**
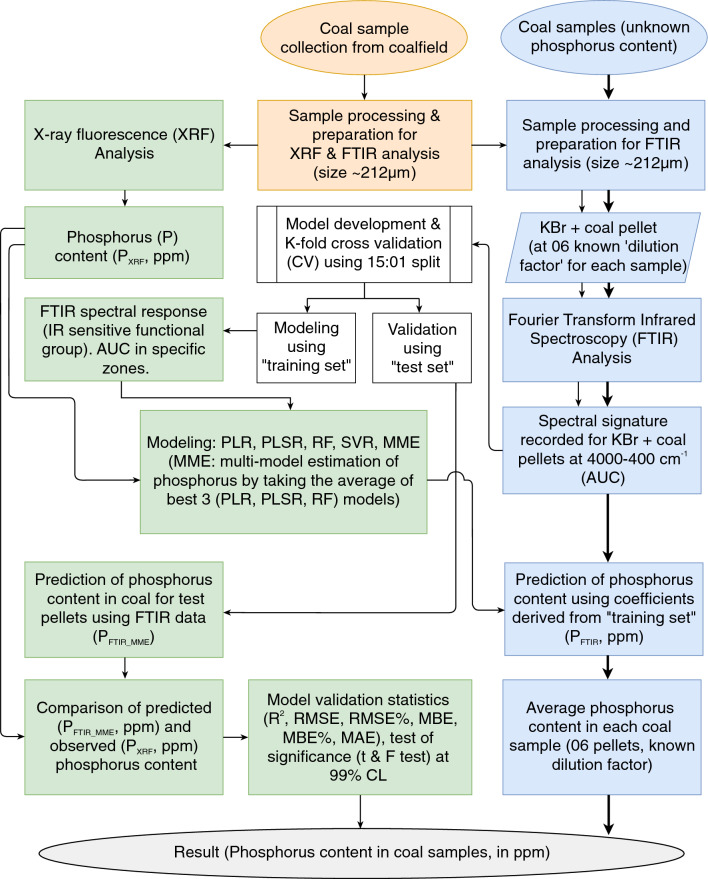
Schematic illustration of the step-by-step procedure used for model development, validation, and estimation of phosphorus content in coal used. The same procedure was repeated for the analysis of phosphorus in coal ash. (AUC, Area under the curve; PLR, piecewise linear regression; PLSR, partial least square regression; RF, random forest, SVR, support vector regression; MME, multi-model estimation).

The present method provides the advantage of detecting low levels of phosphorus in coal, which is particularly crucial for the steel-making industry and environmental regulations. Unlike other methods, it requires no hazardous chemicals, much less sample size, sample preparation, and measurement time. The proposed method using FTIR stands out as a simpler, cost-effective, and rapidly working alternative to measure the phosphorus content in coal and coal ash. The approach, methods, and comparative validation analysis conducted in the study are summarized in Fig. 1.

## Results

### Elemental composition of coal and coal ash

The elemental composition of coal and its ash is determined at various stages of the coal mining process, from exploration and mining to transportation and combustion. This information is utilized to assess the quality and suitability of the coal (for applications such as power generation, steel production, and cement manufacturing) and its ash (for applications such as fertilizers, the construction industry, and contaminant removal). In this study, WDXRF spectroscopy is employed to quantitatively analyze the phosphorus content in coal and coal ash along with sodium, magnesium, aluminium, silicon, potassium, calcium, titanium, manganese, and iron. The phosphorus content in coal and coal ash samples is reported as P_2_O_5_ mass percent, which is converted to phosphorus mass percent using the conversion factor specific to phosphorus. All the concentrations expressed in mass percent were later converted to parts per million (ppm). The phosphorus content analyzed in coal ranges from 215.59 ppm in sample J_01 to 569.96 ppm in sample J_14 while that in coal ash ranges from 135.29 ppm in sample J_01 to 466.97 ppm in sample J_16. A comprehensive report of the WDXRF analysis conducted on coal and coal ash samples is provided in Tables 1 and 2 respectively.

**Table 1:** XRF spectroscopy analysis report providing the elemental composition of coal.

XRF Spectroscopy (mass %)												
Sample	Na_2_O	MgO	Al_2_O_3_	SiO_2_	P_2_O_5_	P (ppm)	K_2_O	CaO	TiO_2_	MnO	Fe_2_O_3_	Total
J_01	0.418	1.428	24.880	43.123	0.049	215.591	0.828	7.240	2.549	0.241	19.243	100.00
J_02	0.000	0.297	21.800	40.473	0.071	308.549	0.439	1.952	2.108	0.144	32.717	100.00
J_03	0.190	1.226	23.819	47.707	0.114	497.520	0.576	10.495	2.680	0.241	12.951	99.99
J_04	0.155	0.424	22.762	55.386	0.087	380.559	0.604	9.829	5.248	0.272	5.233	100.00
J_05	0.159	0.742	19.755	44.277	0.088	385.796	0.405	9.053	2.790	0.333	22.399	100.00
J_06	0.529	0.734	24.213	54.267	0.068	294.584	1.468	7.132	4.460	0.210	6.919	100.00
J_07	0.087	0.614	23.049	62.724	0.103	448.204	1.295	5.578	3.418	0.095	3.038	100.00
J_08	0.065	0.614	21.376	44.862	0.073	318.587	0.555	3.544	2.834	0.254	25.822	99.99
J_09	0.114	0.873	24.418	57.573	0.094	408.926	1.533	5.192	3.885	0.097	6.222	100.00
J_10	0.178	0.374	22.406	59.077	0.090	392.342	0.550	7.935	6.132	0.180	3.078	99.99
J_11	0.122	0.571	20.061	40.503	0.091	397.579	0.308	7.219	2.279	0.263	28.582	100.00
J_12	0.329	1.389	25.293	42.923	0.055	238.286	0.836	6.390	1.975	0.249	20.562	100.00
J_13	0.136	0.892	24.659	56.556	0.102	444.713	0.309	8.598	3.643	0.212	4.895	99.99
J_14	0.289	1.708	23.729	42.479	0.131	569.966	0.695	16.905	3.504	0.373	10.188	99.99
J_15	0.129	0.395	23.431	59.212	0.078	339.099	0.526	7.615	5.511	0.182	2.921	100.00
J_16	0.117	0.809	23.801	52.465	0.126	550.327	0.283	8.355	3.220	0.320	10.504	99.99

**Table 2:** XRF spectroscopy analysis report providing the elemental composition of coal ash.

XRF Spectroscopy (mass %)												
Sample	Na_2_O	MgO	Al_2_O_3_	SiO_2_	P_2_O_5_	P (ppm)	K_2_O	CaO	TiO_2_	MnO	Fe_2_O_3_	Total
J_01	0.836	2.565	29.685	49.670	0.031	135.290	0.458	4.454	1.867	0.099	7.354	97.02
J_02	0.268	1.130	28.836	58.183	0.077	336.044	0.249	1.522	1.930	0.041	17.681	109.92
J_03	0.482	3.050	27.793	53.232	0.066	288.038	0.289	6.059	1.996	0.048	5.123	98.14
J_04	0.363	0.862	27.993	61.903	0.048	209.482	0.180	3.834	2.751	0.018	1.243	99.20
J_05	0.385	1.784	24.371	56.118	0.092	401.507	0.220	3.789	2.390	0.224	9.367	98.74
J_06	0.710	1.382	31.115	60.211	0.043	187.661	0.772	3.630	2.595	0.024	2.506	102.98
J_07	0.382	1.278	29.170	68.258	0.080	349.137	0.601	2.819	1.933	0.000	1.194	105.71
J_08	0.255	1.424	27.653	57.406	0.066	288.038	0.275	2.239	2.080	0.107	11.301	102.80
J_09	0.347	1.828	28.087	62.636	0.087	379.686	0.787	3.129	2.377	0.000	2.949	102.22
J_10	0.390	0.921	26.924	61.757	0.074	322.951	0.222	4.047	3.127	0.011	1.149	98.62
J_11	0.348	1.372	24.315	52.596	0.100	436.421	0.170	4.135	1.894	0.147	13.360	98.44
J_12	0.749	2.698	30.355	50.874	0.031	135.290	0.480	4.078	1.562	0.100	8.842	99.77
J_13	0.313	1.685	30.759	63.053	0.090	392.779	0.071	4.082	1.600	0.042	2.028	103.72
J_14	0.617	4.751	20.735	40.324	0.055	240.031	0.379	10.040	2.090	0.088	2.839	81.92
J_15	0.347	0.853	27.531	61.009	0.045	196.389	0.161	3.491	2.810	0.001	0.961	97.21
J_16	0.334	1.568	30.020	60.791	0.107	466.970	0.071	4.298	1.454	0.116	4.147	102.90

### Selection of MIR bands suitable for P determination

**Figure 2:**
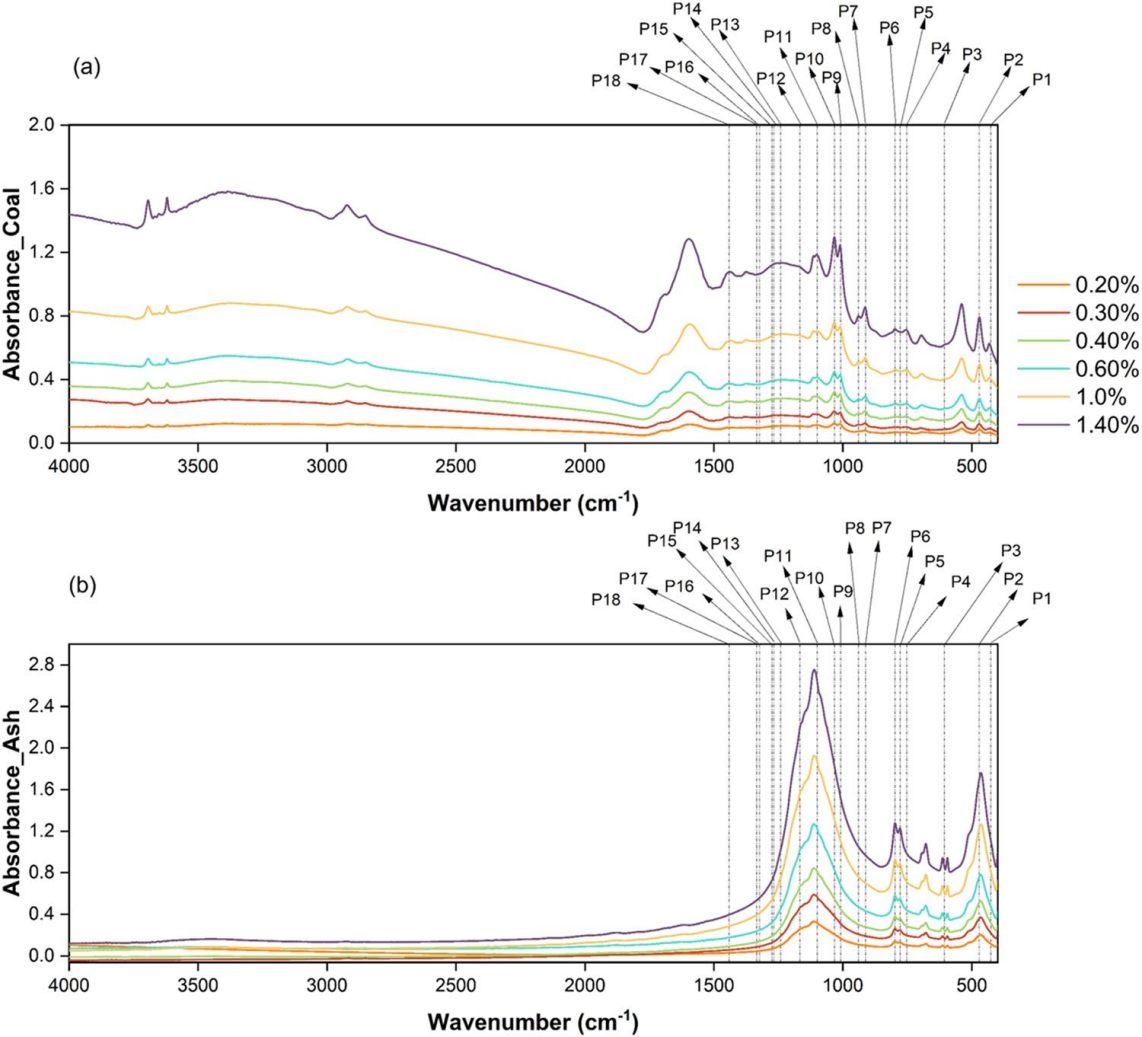
Mid-IR absorbance spectra of (**a**) 06 coal sample pellets and (**b**) 06 coal ash sample pellets of known incremental concentration of sample J_04 showing the identified phosphorus sensitive ranges or peaks as given in table 3.

FTIR is used to identify, analyze and quantify functional groups and chemical bonds in compounds through its ability to measure the absorbance of infrared light at different wavelengths. Phosphorus-containing compounds exhibit characteristic IR absorption bands depending on the specific functional groups present. Phosphorus-containing compounds typically exhibit absorption bands in the IR spectrum at around 1200–950 cm^-1^, which are associated with the stretching vibrations of P–O bonds. Additionally, other functional groups associated with phosphorus, like phosphates, phosphonates, phosphines, phosphine oxides, and phosphate esters, and their infrared active regions, were identified. Besides that, the IR-sensitive frequency range of the PO_4_^3-^ anionic complex common to phosphorus-containing minerals was also identified. A set of 18 peaks was identified and utilized for the current study^44–47^. The 18 mid-IR sensitive peaks of phosphorus for the coal and ash sample J_03 (set of 06 pellets with known sample concentrations showing incremental intensity in absorbance) are shown in Fig. 2. Using the functional group and chemical bond assignments provided in Table 3, the area under the curve (AUC) was determined for all 18 peaks.

**Table 3:** Details of identified MIR absorption peaks of phosphorus compounds and their assigned functional and ionic groups used for the study^44–47^.

Peak	Onset (cm^-1^)	Offset (cm^-1^)	Center (cm^-1^)	Assignment
P_1	419	444	427	O–P–O (scissors deformation)
P_2	444	495	470	
P_3	600	612	607	PO_4_^3-^asymmetric deformation
P_4	730	767	752	P–O (stretch)
P_5	767	787	777	
P_6	787	833	797	
P_7	885	930	912	P–H (bend)
P_8	930	952	939	
P_9	990	1022	1009	P–O–C (asymmetric stretching)
P_10	1022	1065	1032	
P_11	1069	1108	1099	PO_4_^3-^asymmetric valence oscillations
P_12	1140	1191	1165	P=O (stretch)
P_13	1238	1260	1242	
P_14	1260	1272	1268	
P_15	1272	1288	1275	
P_16	1318	1328	1324	
P_17	1332	1338	1334	
P_18	1430	1445	1441	P–C (stretch)

### Model estimation and assessment

For model estimation, a set of six sample pellets was prepared for each of the sixteen coal (coal + KBr) and its ash samples (coal ash + KBr). The six samples were prepared at known dilution factors by fixing the KBr weight at 220 mg and the weight of coal in the sample pellet varying in the order of 0.44, 0.66, 0.88, 1.33, 2.22, and 3.12 mg. The same dilution factors were applied for the preparation of coal ash sample pellets. The spectral response of all 96 coal sample pellets and coal ash sample pellets was recorded individually using Bruker FTIR. The recorded spectra also contained signatures from the KBr spectra, which were mixed with the samples to create pellets. To eliminate the KBr signature from the sample spectra, the spectra of pure KBr pellets were recorded and used as a reference spectrum. This reference spectrum was then subtracted from each pellet’s signature, resulting in a new spectrum that was used for subsequent analysis.

The quantitative analysis of phosphorus content in coal and coal ash samples through FTIR spectroscopy involved the application of piecewise linear regression (P_FTIR_PLR_), partial least square regression (P_FTIR_PLSR_), random forest (P_FTIR_RF_) and support vector regression (P_FTIR_SVR_) models. All models were established with the area under the curve of the eighteen identified absorption peaks associated with the functional groups of phosphorus calculated and used as the independent input variable set. For model development and validation, the K fold cross-validation technique was utilized to test the performance of the FTIR data-based model in determining the phosphorus content in the sample pellets. This cross-validation technique permits the use of samples to build and validate the model using independent sets (“training set” and “test set”) created using a 15:01 split. The K-Fold divides all the samples into k (k = 16) groups of samples. A total of 96 sample pellets for each coal and its ash were used to create 16 groups, where each group had 06 pellets prepared from coal and its ash samples individually at known concentrations. One fold is utilized for testing during each run, while the other folds (K − 1) are used for training so that during the entire process, each fold will be used for testing at least once and is independent of the “training set”.

**Figure 3:**
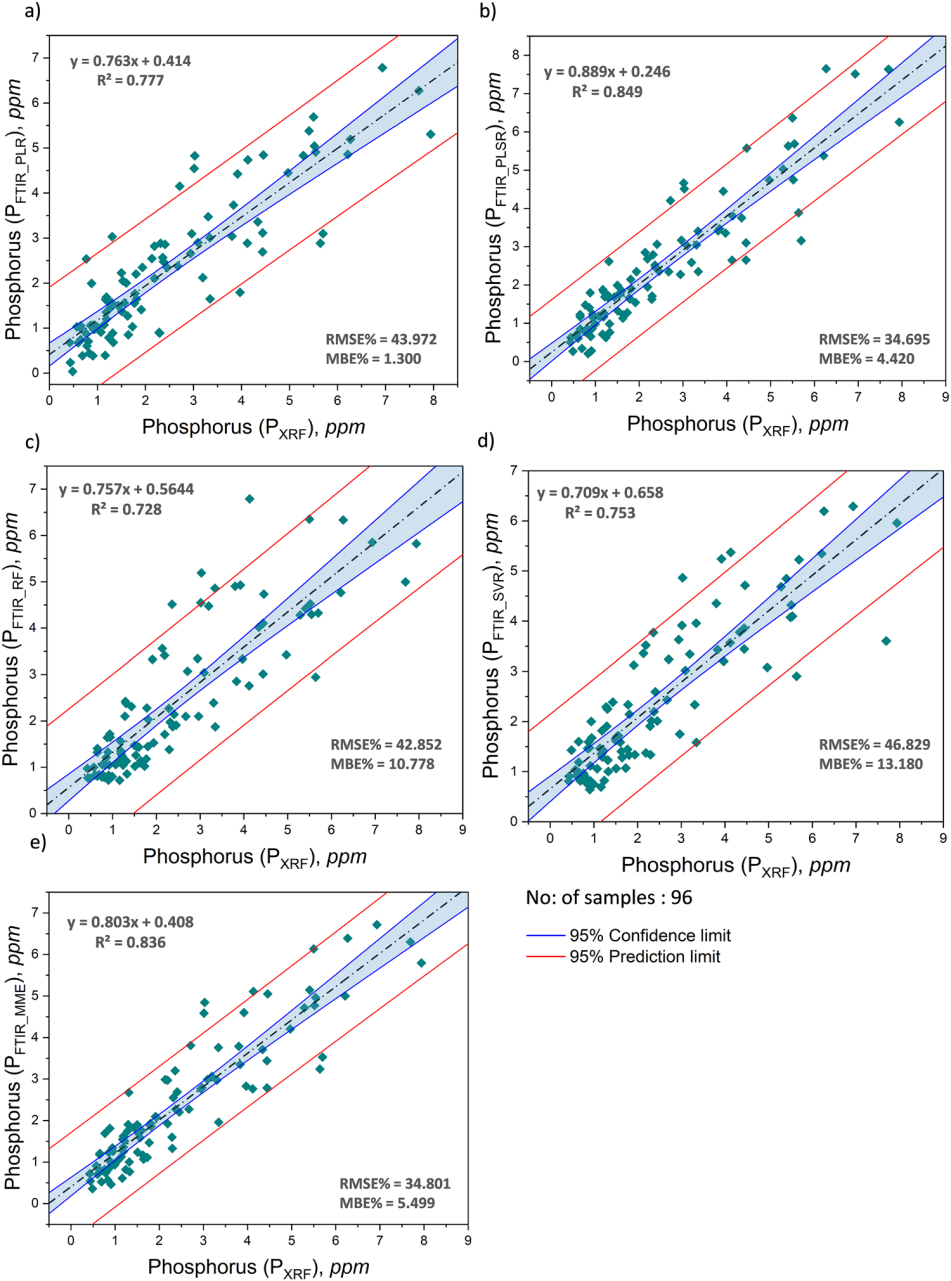
Correlation between phosphorus content in coal measured by XRF (P_XRF_) and modeled using FTIR (**a**) P_FTIR_PLR_ (**b**) P_FTIR_PLSR_ (**c**) P_FTIR_RF_ (**d**) P_FTIR_SVR_ and (**e**) P_FTIR_MME_ using independent “test set” (validation set) using K fold cross-validation.

**Figure 4:**
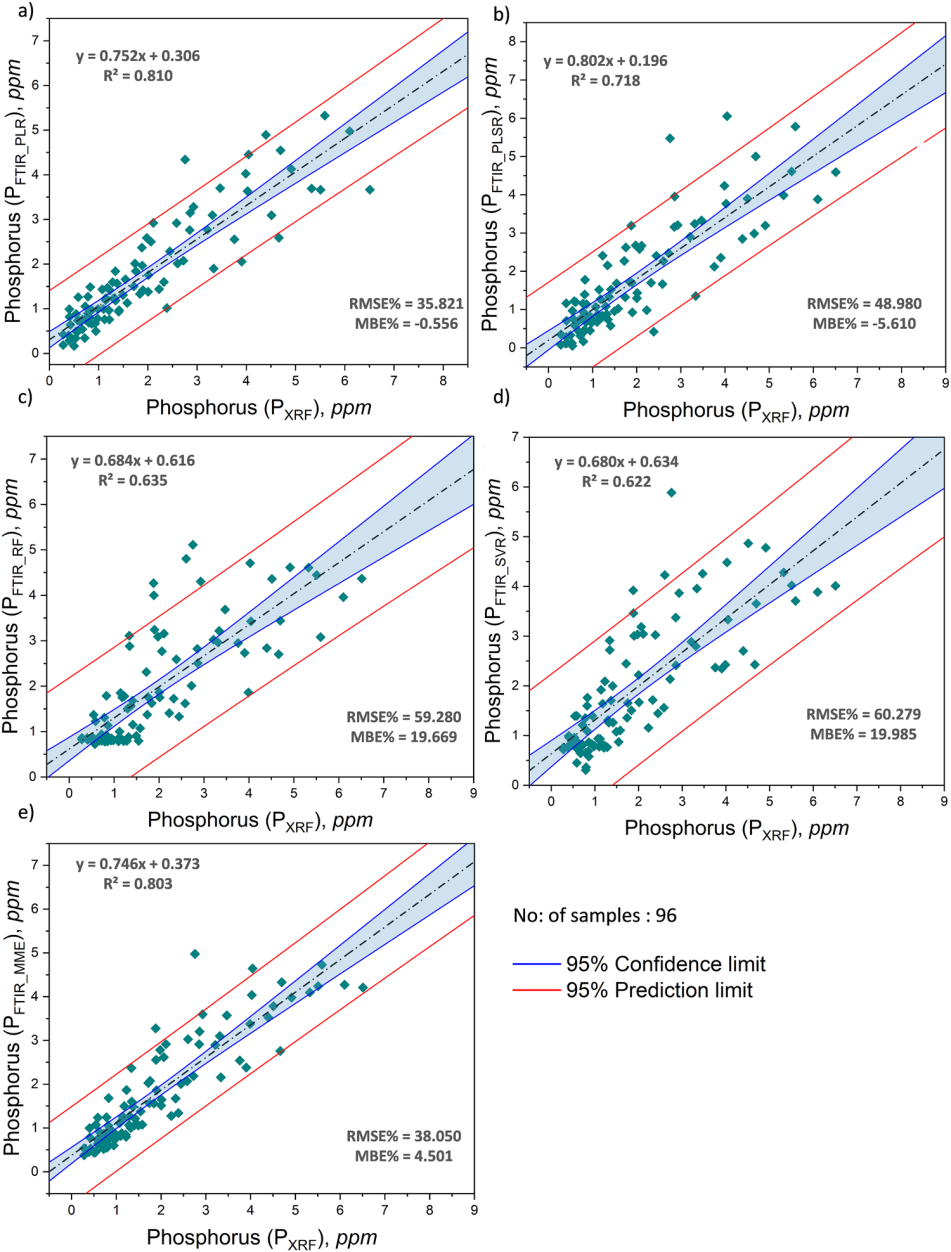
Correlation between phosphorus content in coal ash measured by XRF (P_XRF_) and modeled using FTIR (**a**) P_FTIR_PLR_ (**b**) P_FTIR_PLSR_ (**c**) P_FTIR_RF_ (**d**) P_FTIR_SVR_ and (**e**) P_FTIR_MME_ using independent “test set” (validation set) using K fold cross-validation.

P_FTIR_PLR_, P_FTIR_PLSR_, and P_FTIR_RF_ exhibited enhanced robustness, with predicted values closer to the actual values in the dataset compared to P_FTIR_SVR_. Occasionally, one or more negative values were detected in the PLR and PLSR models, which were replaced by the average of the 3 best performing models. The estimation of phosphorus is found to be more reliable and consistent using the multi-model estimation technique (P_FTIR_MME_) taking the average of the 3 best performing models (P_FTIR_PLR_, P_FTIR_PLSR_, P_FTIR_RF_). The model-estimated phosphorus content for 96 coal samples and 96 coal ash samples can be located in Supplementary Table S1 and Supplementary Table S2, respectively. The model predicted values were compared to the XRF measured phosphorus content to observe the correlation between the two values and the quality of the model (Figs. 3, 4).

**Figure 5:**
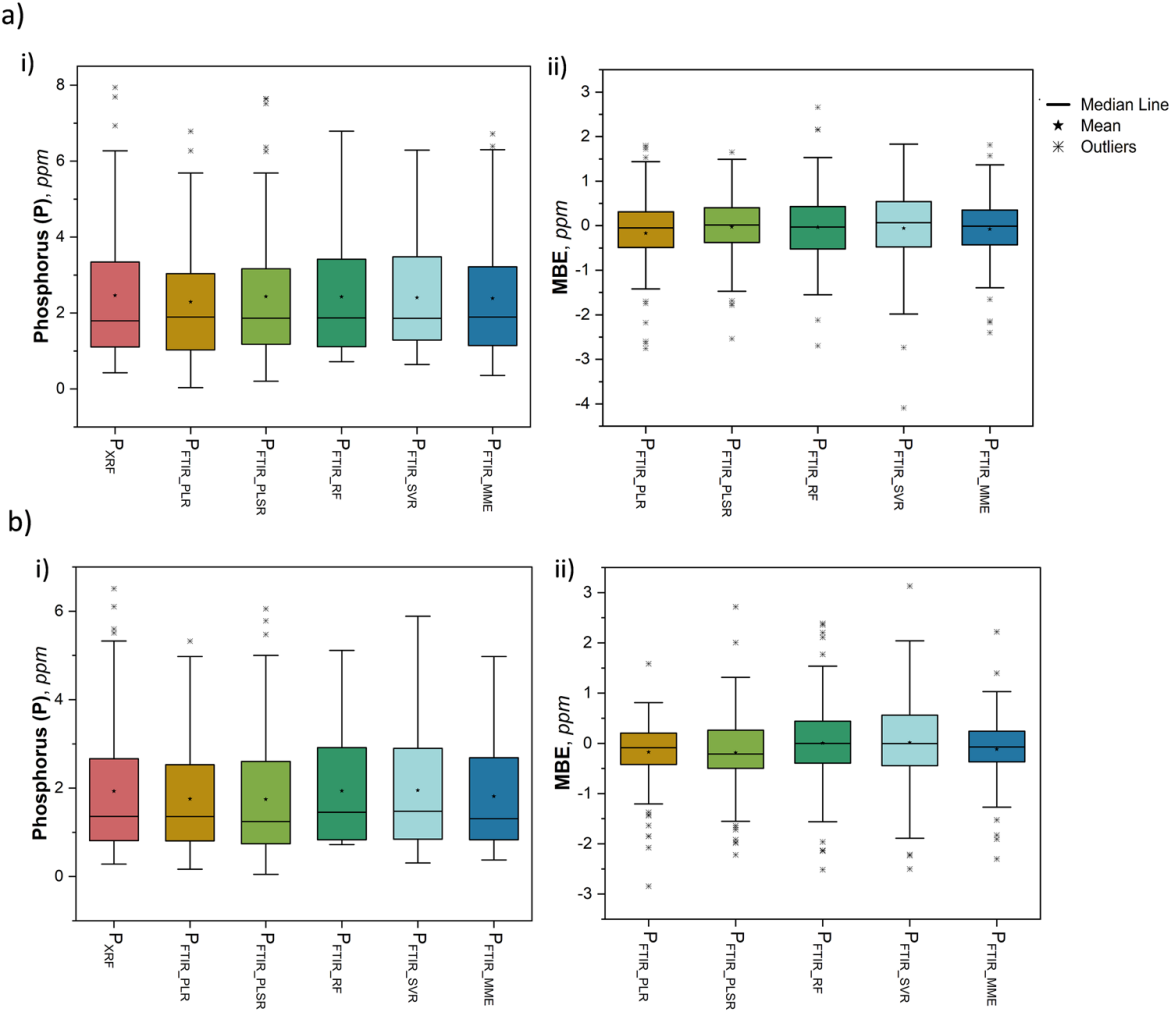
Boxplots corresponding to (**a**) coal i) P content determined in coal by XRF and modeled using FTIR in the experiment, (ii) MBE of the modeled P content in coal using FTIR, and (**b**) coal ash i) phosphorus content determined in ash by XRF and modeled using FTIR in the experiment, (ii) MBE of the modeled phosphorus content in ash using FTIR.

Statistical parameters including the mean ($\mu$), standard deviation ($\sigma$), coefficient of determination (R^2^), root mean squared error (RMSE), mean bias error (MBE), and mean absolute error (MAE) in absolute and percentage are chosen as the performance measures^48^ of the established models. P_FTIR_MME_ showed the highest correlation with P_XRF_ with all R-squared (R^2^) values exceeding 0.80 for both coal and its ash, suggesting a good fit of the model to the data. A comprehensive statistical analysis has been conducted to evaluate the efficacy of the proposed model utilizing the FTIR spectral response of phosphorus functional groups. The comparison between model predicted (P_FTIR_PLR_, P_FTIR_PLSR_, P_FTIR___RF_, P_FTIR_SVR_, P_FTIR_MME_) and XRF measured (P_XRF_) phosphorus content and their correlation for coal and its ash is given in Figs. 3 and 4 respectively. The boxplot representing the distribution of phosphorus determined through XRF and using model predicted FTIR spectroscopy in coal and its ash (Fig. 5a (i),b (i)) shows no significant difference in means. Similarly, the MBE of the presented models was plotted to estimate the average bias in each of the models for coal and ash (Fig. 5a (ii),b (ii)). The MBE of the multi-model estimated phosphorus using FTIR (P_FTIR_MME_) data is found to be low for coal and its ash (− 0.077 ppm and − 0.118 ppm), depicting that both the model performance is consistent and unbiased on average.

Standard statistical significance tests were utilized to test if there is any substantial difference between the means ($\mu$_d_) and variance ($\sigma$^2^) of the phosphorus content obtained from XRF (P_XRF_) and model estimated FTIR spectroscopy (P_FTIR_MME_) of both coal and ash. The paired t-test for means, conducted at a 99% confidence level ($\alpha$ = 0.01), comparing the phosphorus content in coal and its ash measured by XRF and predicted by FTIR yielded p-values greater than $\alpha$=0.01, indicating the inability to reject the null hypothesis (H0: $\mu$_d_ = 0). This suggests no significant difference between the mean values of P_XRF_ and P_FTIR_PLR_, P_FTIR_PLSR_, P_FTIR___RF, _P_FTIR_SVR, _P_FTIR_MME_, in both coal and its ash (Table 4). Similarly, a two-sample F-test for variance at a 99% confidence level also showed *p* value greater than $\alpha$ = 0.01, supporting the acceptance of the null hypothesis (H0: $\sigma$_0_^2^ = $\sigma$p^2^), indicating no significant difference in variance between measured ($\sigma$_0_^2^) and model predicted ($\sigma$_p_^2^) phosphorus values for both coal and coal ash (Table 5). In conclusion, there is no statistically significant difference in mean or variance between measured and predicted phosphorus values at a 99% confidence level for coal and its ash.

**Table 4:** Results of paired two-sample t-test for means (two-tailed at 99 % CI, $\alpha$ = 0.01) to compare the difference in means of measured (P_XRF_) and model estimated P content (P_FTIR_PLR_, P_FTIR_PLSR_, P_FTIR___RF, _P_FTIR_SVR, _P_FTIR_MME_) in (a) coal and (b) coal ash.

Pair	$\mu$	$\sigma$^2^	**t**_**stat**_	**p-value**	**t**_**critical**_	H0: $\mu$_d_ = 0
(a) Coal: t-test: Paired Two Sample for Means, n = 96; df = 95						
P_FTIR_PLR_ (ppm)	2.294	2.446	1.938	0.056	2.629	T
P_FTIR_PLSR_ (ppm)	2.434	3.034	0.394	0.695	2.629	T
P_FTIR_RF_ (ppm)	2.428	2.566	0.362	0.718	2.629	T
P_FTIR_SVR_ (ppm)	2.405	2.181	0.628	0.532	2.629	T
P_FTIR_MME_ (ppm)	2.385	2.518	1.03	0.305	2.629	T
P_XRF_ (ppm)	2.462	3.265				
(b) Ash: t-test: Paired Two Sample for Means, n = 96; df = 95						
P_FTIR_PLR_ (ppm)	1.757	1.552	2.593	0.011	2.629	T
P_FTIR_PLSR_ (ppm)	1.745	1.991	2.268	0.026	2.629	T
P_FTIR_RF_ (ppm)	1.937	1.638	-0.061	0.951	2.629	T
P_FTIR_SVR_ (ppm)	1.948	1.653	-0.176	0.860	2.629	T
P_FTIR_MME_ (ppm)	1.813	1.540	1.733	0.086	2.629	T
P_XRF_ (ppm)	1.931	2.223				

**Figure 6:**
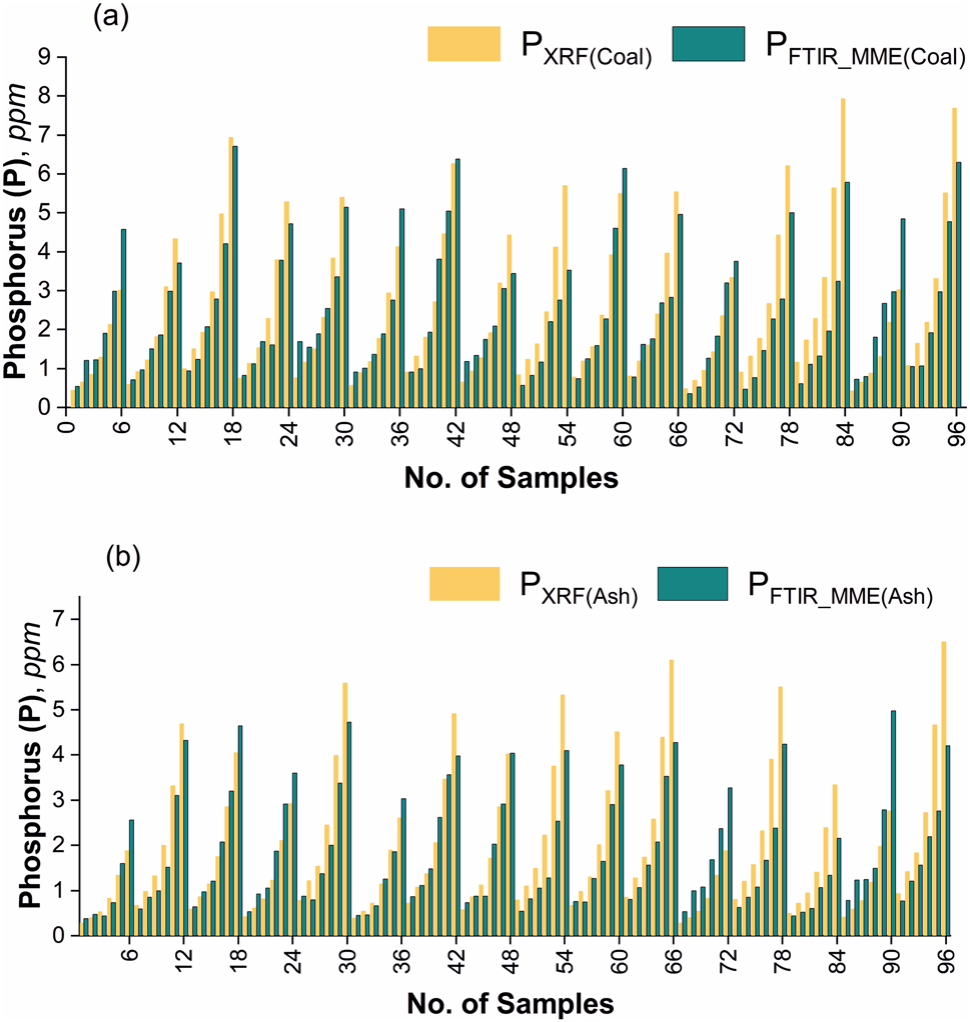
Comparison chart of the P content (ppm) measured by XRF (P_XRF_) and by multi-model FTIR (P_FTIR_MME_) technique for (**a**) coal, and (**b**) coal ash samples.

The comparison between the phosphorus content measured by XRF (P_XRF_) and predicted using multi-model FTIR spectroscopy (P_FTIR_MME_) for coal and its ash samples is given in Fig. 6a,b respectively, which clearly depict that the multi-model estimated phosphorus content is similar and comparable to the measured phosphorus content in both the samples.

**Table 5:** Results of paired two-sample F-test for variance (two-tailed at 99 % CI, $\alpha$ = 0.01) to compare the difference in means of measured (P_XRF_) and model estimated P content (P_FTIR_PLR_, P_FTIR_PLSR_, P_FTIR___RF, _P_FTIR_SVR, _P_FTIR_MME_) in (a) coal and (b) coal ash.

Pair	$\mu$	$\sigma$^2^	**F**_**stat**_	**p-value**	**CI**	H0: $\sigma$_0_^2^ = $\sigma$p^2^
(a) Coal: F-test: Two sample for Variances, n = 96; df = 95						
P_ FTIR_PLR_ (ppm)	2.294	2.446	1.334	0.162	0.783, 2.274	T
P_FTIR_PLSR_ (ppm)	2.434	3.034	1.076	0.723	0.631, 1.833	T
P_FTIR_RF_ (ppm)	2.428	2.566	1.271	0.244	0.746, 2.167	T
P_FTIR_SVR_ (ppm)	2.405	2.181	1.496	0.051	0.878, 2.549	T
P_FTIR_MME_ (ppm)	2.385	2.518	1.297	0.208	0.761, 2.209	T
P_XRF_ (ppm)	2.462	3.265				
(b) Ash: F-test: Two sample for Variances, n = 96; df = 95						
P_FTIR_PLR_ (ppm)	1.757	1.552	1.433	0.081	0.841, 2.442	T
P_FTIR_PLSR_ (ppm)	1.745	1.991	1.117	0.591	0.655, 1.903	T
P_FTIR_RF_ (ppm)	1.937	1.638	1.358	0.138	0.797, 2.314	T
P_FTIR_SVR_ (ppm)	1.948	1.653	1.344	0.151	0.789, 2.290	T
P_FTIR_MME_ (ppm)	1.813	1.540	1.444	0.075	0.847, 2.460	T
P_XRF_ (ppm)	1.931	2.223				

All the results collectively suggest that multi-model FTIR spectroscopy is a novel, sensitive, and reliable alternative to predict low levels of phosphorus content in coal and its ash.

## Discussion

Researchers have investigated several methods to determine the phosphorus content in soils, compound fertilizers, sludge waste, seafood, etc., using different spectroscopic analytical techniques like laser-induced breakdown spectroscopy (LIBS), prompt gamma neutron activation analysis (PGNAA), XRF, X-ray absorption spectroscopy (XAS), ICP-OES, ICP-MS, near-infrared spectroscopy (NIRS), mid-infrared spectroscopy (MIRS), and visible-near infrared spectroscopy (vis-NIRS) (Table 6). PGNAA delivers continuous online monitoring of coal composition but comes with a high cost and radiation hazards. XRF offers immediate analysis capabilities but is limited to detecting elements with atomic numbers lower than 11^49^. NIRS is an established technique utilized in many industries to ensure quality, but the low dipole moment between the phosphorus and oxygen atoms hinders the direct detection of phosphorus or phosphate through NIRS. Nevertheless, the quantification of phosphorus is feasible through NIRS if it is organically bound or closely linked to other soil properties. The limited availability and accuracy of NIRS models for predicting phosphorus content in soil can thus be justified^50,51^. Researchers have employed MIRS to estimate the levels of plant-available phosphorus^50,52,53^. Studies show that MIRS outperformed NIRS, especially for available phosphorus analyses^52^. However, despite these advantages, the calibration functions for both IR methods still had relatively low R^2^ values. The capacity for minimal invasiveness and high-quality soil phosphorus sensing through IR (NIRS and MIRS) spectroscopy is anticipated to improve significantly with the incorporation of more efficient mathematical spectra analysis using machine learning techniques^50^.

**Table 6:** Comparison of the statistical parameter values achieved for models developed in previous studies and present model to estimate P content in different samples (ISAM, Improved Standard Addition Method; FTIR-PAS, Fourier transform mid-infrared photoacoustic spectroscopy; GA-PLS, Genetic algorithm partial least squares; ANN, Artificial neural networks; WET, Water extractable phosphorus; PAP, Plant available phosphorus).

Sl. no.	Method	Nature & sample count	R^2^ RMSE (RMSE%)	MBE (MBE%)	MAE RSD%	Location reference
1	LIBS with linear regression	Soil; N = 90	0.99 NA	NA NA	NA NA	Denmark^74^
2	LIBS with PLSR	Soil; N = 30	0.59 NA	NA NA	NA NA	China^33^
3	LIBS with SVR	Soil; N = 30	0.99 NA	NA NA	184.71 0.0006	China^33^
4	LIBS with linear fitting	Soil; N = 30	0.24 NA	NA NA	NA NA	China^33^
5	LIBS with SVM	Seafood; N = 21	0.99 NA	NA NA	NA 5.18	China^54^
6	LIBS with linear regression	Soil; N = 10	0.86 NA	NA NA	0.0068 NA	China^75^
7	ICP-OES with ISAM	Soil; N = 40	NA NA	NA NA	NA 1.23	China^76^
8	LIBS with PLSR	Soil; N = 147	0.76 NA	1.9 NA	NA NA	Denmark^55^
9	LIBS with linear correlation	Fertilizer; N = 26	0.9 NA	NA NA	NA NA	Brazil^77^
10	vis-NIRS with PLSR	Soil; N = 147	0.55 NA	0.54 NA	NA NA	Denmark^78^
11	LIBS-vis-NIRS with PLSR	Soil; N = 147	0.74 NA	-2.9 NA	NA NA	Denmark^55^
12	vis-NIRS with PLSR	Soil; N = 60	0.91 0.16	NA NA	NA NA	China^78^
13	FTIR-PAS with PLSR (for WEP)	Soil; N = 60	0.80 0.78	NA NA	NA NA	Denmark^79^
14	FTIR-PAS with PLSR (for PAP)	Soil; N = 60	0.7 134.1	NA NA	NA NA	Denmark^79^
15	Vis-NIRS with GA-PLS	Soil; N = 103	0.79 NA	NA NA	NA NA	Japan^80^
16	Vis-NIRS with ANN	Soil; N = 41	0.81 NA	NA NA	NA NA	Thailand^53^
17	FTIR with PLR	Coal; N = 96	0.777 0.865 (43.972)	− 0.169 (1.30)	0.6 NA	Present study India
		Coal ash; N = 96	0.810 0.677 (35.821)	− 0.174 (− 0.556)	0.450 NA	
18	FTIR with PLSR	Coal; N = 96	0.849 0.703 (34.695)	− 0.028 (4.420)	0.511 NA	
		Coal ash; N = 96	0.718 0.822 (48.980)	− 0.186 (− 5.610)	0.596 NA	
19	FTIR with RF	Coal; N = 96	0.728 0.940 (42.852)	− 0.035 (10.778)	0.701 NA	
		Coal ash; N = 96	0.635 0.901 (59.280)	0.006 (19.669)	0.639 NA	
20	FTIR with SVR	Coal; N = 96	0.753 0.900 (46.829)	− 0.058 (13.180)	0.657 NA	
		Coal ash; N = 96	0.622 0.918 (60.279)	0.017 (19.985)	0.696 NA	
21	FTIR with MME (multi-model estimation using PLR,PLSR,RF)	Coal; N = 96	0.836 0.735 (34.801)	− 0.077 (5.500)	0.528 NA	
		Coal ash; N = 96	0.803 0.676 (38.050)	− 0.118 (4.501)	0.474 NA	

In the field of prediction modeling, several statistical evaluation metrics are commonly used to evaluate the quality of these models. These metrics include the correlation between measured and predicted values like R^2^, RMSE, MBE, and MAE. In general, models with higher R^2^ and lower RMSE and MBE values are considered to have better prediction quality. It is commonly accepted that models with an R^2^ value greater than 0.70 with lower MAE and RMSE are deemed to have acceptable predictive power, while models with an R^2^ value below 0.50 and a relatively higher MAE and RMSE value are considered to have poor prediction capability^50^.

It can be noticed from Table 6 that a few models using LIBS with multivariate analysis based on machine learning achieve good prediction capability for phosphorus content in the soil compared to other methods. The literature review shows that LIBS with the SVM prediction model provides the best results in soil samples. However, the diverse characteristics of soil samples, including their heterogeneity, roughness, and particle size, create uncertainties when implementing LIBS, particularly due to the matrix effect impacting quantification accuracy^54,55^. LIBS technology has been used to identify and quantify several major elements (C, H, O, S, Si) along with very few minor elements (Fe, Ti, Al, Ca, Na, Cu)^28,49,56–58^. To the best of our knowledge, no substantial literature or models estimate the phosphorus content of coal and ash using mid-infrared FTIR spectroscopy. Thus, the present attempt to determine the phosphorus content of coal and ash using mid-infrared FTIR spectroscopy is a novel method. Table 6 shows that the multi-model estimation of phosphorus proposed in this study using FTIR data for coal samples presents a good R^2^ of 0.835 and R^2^ of 0.803 for the coal ash samples (with relatively low RMSE and MBE) and is comparable to other models reviewed. Compared to FTIR, LIBS tends to be relatively more expensive and complex due to its intricate laser systems, leading to higher initial purchase expenses. Additionally, maintenance and calibration needs are more frequent in LIBS due to the complexities associated with laser operation and plasma generation. Therefore, the proposed model using FTIR with multivariate analysis based on machine learning techniques shows promising potential as a real-time, cost-effective alternative analytical approach for rapidly and accurately quantifying phosphorus in coal and coal ash. The model’s accuracy can be further enhanced by incorporating a greater number of samples from other coal field basins. The model presented here may be developed further to facilitate improvements in the monitoring and analysis of phosphorus content in coal and ash. Phosphorus content in a variety of other natural materials, such as soil, shale, and others, can be potentially estimated by making suitable adjustments to the mid-infrared FTIR data-based model.

## Methods

### Coal and ash samples

All the coal samples used in the study were collected from the Johilla coalfield of the Son-Mahanadi Basin, present in the Umaria district of Madhya Pradesh, India. The samples were collected from five underground projects at Kundri, Pali, Pinoura, Umaria, Vindhya, and one opencast project at Kanchan in the coalfield, as per ASTM D-2234^59^ guidelines. A total of sixteen samples used in this study were collected from each of these locations. The collected coal samples were crushed and sieved to a size of 212 $\upmu$m following the ASTM D-4749^60^ standard procedure for XRF and FTIR analysis. As per the ASTM standard test method, 1 g of the same samples (212 $\upmu$m) was weighted and combusted at temperatures of $750\,^{\circ }\hbox {C} \pm 10\,^{\circ }\hbox {C}$ for 1 hour for ash yield^61^.

### X-ray fluorescence spectroscopy

The X-ray fluorescence spectroscopy of whole coal samples and their coal ash was conducted in the Rigaku ZSX Primus IV Wavelength Dispersive X-Ray Fluorescence (WDXRF) spectroscopy at the Central Research Facility, Indian Institute of Technology (Indian School of Mines) Dhanbad (IIT(ISM) Dhanbad), Jharkhand, India. The analysis of coal samples has the advantage of being able to detect volatile elements that may be lost during ashing or fusion. A tube-above sequential wavelength dispersive X-ray fluorescence (WDXRF) spectrometer is used to determine the concentrations of major and minor atomic elements in samples. The accuracy of the WDXRF spectrometric measurements of elements depends on various factors such as concentration, particle size, matrix effects, surface roughness, and the quality of standard materials. The samples were filled in an aluminium mold and pressed for roughly one minute at 200 kN to prepare pellets. In this study, the WDXRF technique was used to determine the total phosphorus concentration, which served as the reference for phosphorus levels.

### Fourier-transform infrared (FTIR) spectroscopy

FTIR spectroscopy is the most common form of infrared spectroscopy used for identifying the vibrations of functional groups and anionic groups. The FTIR spectra of the coal and coal ash samples were recorded using an INVENIO S, BRUKER OPTIK, GmBH (model & make) at the Department of Applied Geology, IIT(ISM) Dhanbad, Jharkhand, India. Specimen pellet preparation for FTIR analysis involves weighing and homogenously mixing the ground sample powder (212 $\upmu$m) with potassium bromide (KBr) powder (IR spectroscopy grade, Uvasol, Kaliumbromid, Germany). This composite (coal + KBr & coal ash + KBr) is filled on the anvil surface of the KBr die and spread by inserting the plunger. The assembled die was placed on the cover plate of the cylinder of the hydraulic press, and pressure was applied ($\tilde{6}$ tons for 5 min) to make circular, thin pellets of diameter 1.3 cm. To reduce the risk of moisture and other gases affecting the experiment, the FTIR optical bench was purged with N_2_ gas flowing at 200 l per hour for a duration of 2 h before conducting the analysis. The KBr pellets that had been prepared were placed in a quick lock base plate positioned in the FTIR sample chamber. The sample pellets were then subjected to infrared radiation, allowing for the measurement of the resulting absorption spectrum. Subsequently, the absorbance versus frequency plot (Y–X plot) was generated based on the acquired spectra. For the coal samples, the absorption spectra were limited to the wavenumber range of 4000–400 cm^-1^.

### Piecewise linear regression (PLR)

The estimation model has been used to predict the phosphorus content in coal and coal ash by computing the relationship between a set of independent variables (derived from the FTIR spectra) and a dependent variable (observed P content from XRF). A piecewise linear empirical equation with a breakpoint and quasi-newton method, along with a least squares loss function, was used to solve the coefficients of the model using the training data^42^. Through the iterative convergence of an empirical equation that has been predefined, this non-linear method can be utilised to achieve the goal of minimizing the least square’s function. The coefficients of the empirical equation are typically reliant on the pool of input data that is available^62^. To achieve optimal values for the coefficients, the process of optimization could involve numerous iterative convergences on the empirical equation and the data that was chosen. It is possible to compute the loss function in such a way that, at each iteration, the goal is to reduce the square of the difference between the observed and predicted phosphorus content using an empirical equation that has been predefined^63^. The optimization method utilizes the 1st order derivative of a function to determine the function’s slope at a given point and the 2nd order derivative to determine the rate and direction of change of the slope. This method evaluates the function at different points during each step to approximate the first- and second-order derivatives, further utilized to identify the minimum loss function. By minimizing the sum of squared errors between observed data and model predictions, the method can estimate statistical model parameters for estimation problems^64^.

When analyzing with a breakpoint in the model, two sets of coefficients are produced for the variables (QNbp_L and QNbp_R where bp_L and bp_R are model generated with breakpoint corresponding to the left and right equations, respectively. A single form of the coefficient (QNnbp) was obtained for the model with no breakpoint. QNbp(avg) is also found by averaging the phosphorus content obtained from the left and right equations. Notably, the estimated value of phosphorus (ppm) in coal obtained from QNnbp using the no breakpoint is often the closest estimate to the actual experimental value. At times, the non-breakpoint estimated phosphorus (ppm) values are outside the expected range of ± 1.5 IQR (inter-quartile range). To address this issue, the interquartile range (IQR) was determined to identify and eliminate any out-of-range values. The proposed model works on the following conditions: the value from the QNnbp model is taken into consideration if the projected phosphorus content from QNnbp is between the low and high ranges (Q1 − 1.5 IQR or Q3 + 1.5 IQR, respectively). Else, if the modeled phosphorus content in coal from QNnbp is beyond the low or high range (Q1 − 1.5 IQR or Q3 + 1.5 IQR, respectively), the *P* value obtained from the QNbp_avg model is taken into consideration.

In the case of ash samples, the model with a breakpoint corresponding to the left equation (QNbp_L) provides the closest estimate to the actual experimental value of phosphorus in ash. If the projected phosphorus content in ash from QNbp_L is not between the low and high ranges (Q1 − 1.5 IQR or Q3 + 1.5 IQR, respectively), the *P* value obtained from QNnbp model is taken into consideration. The accuracy of phosphorus content estimation in coal using PLR is improved by this method of defining a threshold to identify out-of-range results. It helps to restrict errors in the estimation of phosphorus content in unknown samples.

### Partial least square regression (PLSR)

Partial least squares, also referred to as “projection to Latent Structures,” is a category of learning techniques developed to model the relationship of observed variables using latent variables. PLSR calculates components by maximizing covariance between feature and response matrices, making it particularly effective for problems featuring many highly correlated features and multiple responses. Developed for predicting, PLSR finds application in various spectroscopies, such as near-infrared reflectance (NIR) spectroscopy, Fourier transform infrared (FTIR) spectroscopy, and Fourier transform-Raman (FT-Raman) spectroscopy^65^.

Implementation of PLSR involves simultaneous decomposition of the spectral matrix and concentration matrix to eliminate redundant information, fully considering their relationship to derive an optimized calibration model. The outcome is a linear relationship establishing the basis for quantitative analysis of material elements^66^. PLSR resembles principal component regression but differs in using target variables to identify scores highly covariant with predictor variables. PLSR excels with datasets demonstrating the multicollinearity of predictor variables, and its advantage over PCR lies in the reduced optimal number of components^67,68^. The model is created using the cross_decomposition module of the scikit-learn library in the Python programming environment.

### Random forest (RF)

Random forest is a supervised ensemble learning method employed for both regression and classification tasks. Ensemble learning integrates predictions from multiple models to yield more accurate results than a single model. It produces numerous decision trees and develops their predictions to make final predictions. Decision trees are fundamental models that predict outcomes by executing splits based on predictors that maximally reduce mean squared error^69^. The approach is rooted in integrating multiple decision trees, offering robustness to non-linearity, and is best suited for modeling nonlinear data. The combination of individual decision trees mitigates their high variance, addressing the overfitting issue without requiring pruning^70,71^. In this study, a random forest regressor model from the ensemble module of the scikit-learn library in the Python programming environment is utilized. Hyperparameter tuning is conducted through RandomizedSearchCV and GridSearchCV. After comparing with the default hyperparameter settings, the best estimator is selected for modeling.

### Support vector regression (SVR)

Support Vector Machine (SVM) is a robust supervised machine learning paradigm rooted in a multivariate nonlinear correction approach introduced in the 1990s with a problem-solving capacity involving nonlinearity and high dimensionality. SVM has evolved through ongoing algorithmic optimization, showcasing impressive learning performance in nonlinear regression and function approximation, leading to widespread adoption in quantitative research, especially in spectral analysis, and the development of support vector regression^33,65^. The model simplifies conventional regression processes by efficiently predicting outcomes on training data using a technique known as “transduction inference”^72^. Its core function involves identifying the optimal hyperplane that separates data in a multi-dimensional space, minimizing errors across all training samples^73^. For model optimization and training, the GridSearchCV function and the SVM module within the scikit-learn library in the Python programming environment were employed.

## Conclusions

Accurate and rapid measurement of phosphorus content in coal and coal ash is crucial for efficient and sustainable utilization. In this investigation, we evaluated the ability of Fourier transform infrared spectroscopy (FTIR) in conjunction with machine learning models like piecewise linear regression (PLR), partial least square regression (PLSR), random forest (RF), and support vector regression (SVR) for quantifying the phosphorus content in coal and coal ash. The study proposes a novel FTIR-based multi-model approach combining PLR, PLSR, and RF regression models to be a reliable alternative to traditional analytical methods for the rapid and near real-time measurement of phosphorus in coal and coal ash. The major findings of the study are:

- The FTIR-based model is sensitive enough to estimate very low levels of phosphorus in both coal and coal ash samples.
- PLR, PLSR, and RF methods exhibited enhanced robustness compared to the SVR method. The estimation of phosphorus is found to be more consistent using a multi-model estimation technique (FTIR_MME), taking the average of the three best-performing models. The accuracy of the proposed model to estimate the phosphorus content in coal and ash is relatively good (R$^2$ of 0.836, RMSE of 0.735 ppm, RMSE (%) of 34.801, MBE of − 0.077 ppm, MBE (%) of 5.499, and MAE of 0.528 ppm in coal samples, and R$^2$ of 0.803, RMSE of 0.676 ppm, RMSE (%) of 38.050, MBE of − 0.118 ppm, MBE (%) of 4.501, and MAE of 0.474 ppm in coal ash samples).
- The determination of phosphorus in coal and coal ash using mid-infrared FTIR (P_FTIR_MME_) is a promising cost-effective alternative compared to conventional methods such as XRF (P_XRF_).
- Statistical tests of significance using two-tailed paired t-test for means and F-test for variances prove that there is no difference in means and variances, respectively, between XRF measured phosphorus content (P_XRF_) and multi-model-estimated phosphorus content (P_FTIR_MME_) in coal and coal ash.

The model’s accuracy can be further enhanced by incorporating a greater number of samples from other coal field basins. The model presented here may be developed further to facilitate improvements in the monitoring and analysis of phosphorus content in coal and ash. Phosphorus content in a variety of other natural materials, such as soil, shale, and others, can be potentially estimated by making suitable adjustments to the mid-infrared FTIR-data based model.

## Supplementary Information

Supplementary Information.

## Data Availability

All data generated or analyzed during this study are included in this published article (and its Supplementary Information files).
