# Supplementary material for: A novel multi-model estimation of phosphorus in coal and its ash using FTIR spectroscopy
Source: Sci Rep. 2024 Jun 14;14:13785. doi: 10.1038/s41598-024-63672-x (PMC11637066; doi:10.1038/s41598-024-63672-x)
Supplement: Supplementary file 1 — Supplementary Information. [file 41598_2024_63672_MOESM1_ESM.zip › Supplementary Information (SI).docx]

**A novel multi-model estimation of phosphorus in coal**

**and its ash using FTIR spectroscopy**

**Arya Vinod**1**, Anup Krishna Prasad**1,4***, Sameeksha Mishra**1**, Bitan Purkait**1**, Shailayee**

**Mukherjee**1,4**, Anubhav Shukla**1,2**, Nirasindhu Desinayak**3**, Bhabesh Chandra Sarkar**4**, and**

**Atul K. Varma**2

1Photogeology and Image Processing Laboratory, Department of Applied Geology, Indian Institute of Technology

(Indian School of Mines), Dhanbad 826004, India; anup@iitism.ac.in

2Coal Geology and Organic Petrology Laboratory, Department of Applied Geology, Indian Institute of Technology

(Indian School of Mines), Dhanbad 826004, India; atul@iitism.ac.in

3Department of Geology, Ravenshaw University, Cuttack, Odisha, 753003, India;

nirasindhu@ravenshawuniversity.ac.in

4Geocomputational and GIS Laboratory, Department of Applied Geology, Indian Institute of Technology (Indian

School of Mines), Dhanbad 826004, India

*anup@iitism.ac.in

**Supplementary Table S1:** Comparison table of the phosphorus content (ppm) measured by XRF (P_XRF_) and by FTIR techniques (P_FTIR_PLR_, P_FTIR_PLSR_, P_FTIR_RF_, P_FTIR_SVR_, P_FTIR_MME_) in coal.

| Sample No. | P_XRF_ | P_FTIR_PLR_ | P_FTIR_PLSR_ | P_FTIR_RF_ | P_FTIR_SVR_ | _PFTIR_MME_ |
| --- | --- | --- | --- | --- | --- | --- |
| 1 | 0.438 | 0.237 | 0.625 | 0.769 | 0.908 | 0.544 |
| 2 | 0.654 | 1.052 | 1.170 | 1.405 | 1.186 | 1.209 |
| 3 | 0.859 | 1.059 | 1.407 | 1.210 | 1.462 | 1.225 |
| 4 | 1.295 | 1.569 | 1.720 | 2.422 | 1.906 | 1.904 |
| 5 | 2.136 | 2.546 | 2.851 | 3.559 | 3.358 | 2.985 |
| 6 | 3.015 | 4.545 | 4.664 | 4.547 | 3.913 | 4.585 |
| 7 | 0.602 | 0.677 | 0.606 | 0.831 | 0.920 | 0.705 |
| 8 | 0.923 | 0.975 | 0.860 | 1.049 | 1.115 | 0.961 |
| 9 | 1.216 | 1.586 | 1.692 | 1.248 | 1.422 | 1.509 |
| 10 | 1.814 | 1.646 | 1.906 | 2.033 | 2.145 | 1.862 |
| 11 | 3.094 | 2.901 | 3.026 | 3.040 | 3.017 | 2.989 |
| 12 | 4.341 | 3.359 | 3.754 | 4.017 | 3.779 | 3.710 |
| 13 | 0.994 | 1.025 | 0.982 | 0.797 | 0.750 | 0.935 |
| 14 | 1.510 | 1.371 | 1.323 | 1.021 | 1.063 | 1.238 |
| 15 | 1.938 | 2.348 | 2.350 | 1.527 | 1.338 | 2.075 |
| 16 | 2.966 | 3.098 | 3.168 | 2.099 | 1.750 | 2.788 |
| 17 | 4.971 | 4.448 | 4.743 | 3.421 | 3.080 | 4.204 |
| 18 | 6.934 | 6.785 | 7.514 | 5.853 | 6.289 | 6.717 |
| 19 | 0.760 | 0.884 | 0.785 | 0.790 | 0.966 | 0.820 |
| 20 | 1.138 | 1.072 | 1.190 | 1.110 | 1.466 | 1.124 |
| 21 | 1.533 | 2.050 | 1.757 | 1.258 | 1.703 | 1.688 |
| 22 | 2.287 | 0.903 | 1.627 | 2.269 | 1.908 | 1.600 |
| 23 | 3.803 | 3.042 | 3.415 | 4.903 | 4.352 | 3.787 |
| 24 | 5.288 | 4.833 | 5.028 | 4.280 | 4.687 | 4.714 |
| 25 | 0.770 | 2.536 | 1.478 | 1.057 | 0.868 | 1.690 |
| 26 | 1.171 | 1.639 | 1.878 | 1.121 | 1.352 | 1.546 |
| 27 | 1.502 | 2.225 | 1.992 | 1.471 | 1.620 | 1.896 |
| 28 | 2.318 | 2.882 | 2.792 | 1.976 | 2.016 | 2.550 |
| 29 | 3.833 | 3.734 | 3.468 | 2.854 | 3.431 | 3.352 |
| 30 | 5.407 | 5.380 | 5.632 | 4.422 | 4.848 | 5.145 |
| 31 | 0.574 | 1.029 | 0.709 | 0.997 | 1.032 | 0.912 |
| 32 | 0.894 | 1.061 | 0.654 | 1.326 | 1.474 | 1.014 |
| 33 | 1.187 | 1.355 | 1.170 | 1.561 | 1.816 | 1.362 |
| 34 | 1.784 | 1.769 | 1.637 | 2.279 | 2.336 | 1.895 |
| 35 | 2.942 | 2.659 | 2.274 | 3.342 | 3.629 | 2.759 |
| 36 | 4.133 | 4.736 | 3.805 | 6.790 | 5.371 | 5.110 |
| 37 | 0.914 | 1.028 | 0.858 | 0.826 | 0.817 | 0.904 |
| 38 | 1.320 | 0.889 | 0.984 | 1.134 | 1.231 | 1.002 |
| 39 | 1.805 | 2.201 | 2.145 | 1.449 | 1.331 | 1.932 |
| 40 | 2.713 | 4.150 | 4.204 | 3.071 | 3.240 | 3.809 |
| 41 | 4.458 | 4.844 | 5.572 | 4.734 | 4.713 | 5.050 |
| 42 | 6.269 | 5.192 | 7.647 | 6.334 | 6.193 | 6.391 |
| 43 | 0.650 | 1.000 | 1.226 | 1.320 | 1.598 | 1.182 |
| 44 | 0.939 | 1.069 | 1.239 | 1.716 | 2.001 | 1.341 |
| 45 | 1.284 | 1.466 | 1.680 | 2.101 | 2.292 | 1.749 |
| 46 | 1.914 | 1.409 | 1.543 | 3.326 | 3.127 | 2.093 |
| 47 | 3.195 | 2.122 | 2.587 | 4.476 | 3.339 | 3.062 |
| 48 | 4.441 | 3.116 | 3.100 | 4.102 | 3.857 | 3.439 |
| 49 | 0.835 | 0.441 | 0.206 | 1.045 | 0.769 | 0.564 |
| 50 | 1.240 | 0.788 | 0.627 | 1.045 | 0.822 | 0.820 |
| 51 | 1.629 | 1.270 | 1.133 | 1.094 | 1.403 | 1.166 |
| 52 | 2.453 | 2.347 | 2.356 | 1.909 | 2.011 | 2.204 |
| 53 | 4.121 | 2.885 | 2.648 | 2.759 | 3.566 | 2.764 |
| 54 | 5.699 | 3.099 | 3.160 | 4.325 | 5.227 | 3.528 |
| 55 | 0.783 | 0.606 | 0.768 | 0.832 | 0.918 | 0.735 |
| 56 | 1.191 | 1.203 | 1.268 | 1.300 | 1.293 | 1.257 |
| 57 | 1.563 | 1.559 | 1.761 | 1.446 | 1.717 | 1.589 |
| 58 | 2.375 | 2.477 | 2.460 | 1.876 | 2.198 | 2.271 |
| 59 | 3.920 | 4.424 | 4.448 | 4.928 | 5.240 | 4.600 |
| 60 | 5.500 | 5.689 | 6.365 | 6.352 | 4.075 | 6.135 |
| 61 | 0.811 | 0.706 | 0.861 | 0.783 | 0.855 | 0.783 |
| 62 | 1.188 | 1.689 | 1.827 | 1.354 | 1.371 | 1.623 |
| 63 | 1.602 | 2.002 | 1.849 | 1.435 | 1.587 | 1.762 |
| 64 | 2.408 | 2.861 | 3.063 | 2.142 | 2.592 | 2.689 |
| 65 | 3.971 | 1.793 | 3.358 | 3.335 | 3.201 | 2.828 |
| 66 | 5.544 | 4.910 | 5.686 | 4.294 | 4.095 | 4.963 |
| 67 | 0.486 | 0.036 | 0.267 | 0.764 | 1.430 | 0.355 |
| 68 | 0.691 | 0.386 | 0.344 | 0.837 | 0.818 | 0.522 |
| 69 | 0.949 | 0.981 | 1.149 | 1.649 | 1.673 | 1.260 |
| 70 | 1.432 | 1.500 | 1.686 | 2.312 | 2.388 | 1.833 |
| 71 | 2.360 | 2.568 | 2.516 | 4.514 | 3.770 | 3.199 |
| 72 | 3.342 | 3.008 | 3.404 | 4.858 | 3.956 | 3.757 |
| 73 | 0.908 | 0.386 | 0.280 | 0.721 | 0.643 | 0.462 |
| 74 | 1.329 | 0.681 | 0.765 | 0.855 | 1.002 | 0.767 |
| 75 | 1.772 | 1.556 | 1.666 | 1.178 | 1.444 | 1.467 |
| 76 | 2.674 | 2.378 | 2.347 | 2.099 | 2.432 | 2.275 |
| 77 | 4.441 | 2.692 | 2.652 | 3.012 | 3.444 | 2.785 |
| 78 | 6.214 | 4.858 | 5.376 | 4.766 | 5.347 | 5.000 |
| 79 | 1.163 | 0.395 | 0.714 | 0.719 | 0.693 | 0.609 |
| 80 | 1.729 | 1.034 | 1.279 | 1.023 | 1.071 | 1.112 |
| 81 | 2.296 | 0.891 | 1.711 | 1.382 | 1.338 | 1.328 |
| 82 | 3.347 | 1.649 | 2.351 | 1.872 | 1.583 | 1.957 |
| 83 | 5.642 | 2.885 | 3.886 | 2.946 | 2.904 | 3.239 |
| 84 | 7.941 | 5.304 | 6.255 | 5.822 | 5.958 | 5.794 |
| 85 | 0.426 | 0.681 | 0.502 | 0.974 | 0.809 | 0.719 |
| 86 | 0.653 | 0.746 | 0.848 | 0.809 | 0.984 | 0.801 |
| 87 | 0.880 | 1.994 | 1.798 | 1.640 | 1.593 | 1.811 |
| 88 | 1.310 | 3.031 | 2.615 | 2.377 | 2.252 | 2.674 |
| 89 | 2.189 | 2.822 | 2.682 | 3.411 | 3.518 | 2.971 |
| 90 | 3.030 | 4.829 | 4.510 | 5.191 | 4.864 | 4.843 |
| 91 | 1.074 | 0.910 | 1.187 | 1.056 | 1.058 | 1.051 |
| 92 | 1.646 | 0.852 | 1.220 | 1.138 | 1.281 | 1.070 |
| 93 | 2.192 | 2.104 | 1.950 | 1.712 | 1.373 | 1.922 |
| 94 | 3.309 | 3.474 | 3.048 | 2.392 | 2.338 | 2.971 |
| 95 | 5.520 | 5.044 | 4.748 | 4.522 | 4.319 | 4.771 |
| 96 | 7.692 | 6.273 | 7.636 | 4.997 | 3.601 | 6.302 |

**Supplementary Table S2:** Comparison table of the phosphorus content (ppm) measured by XRF (P_XRF_) and by FTIR techniques (P_FTIR_PLR_, P_FTIR_PLSR_, P_FTIR_RF_, P_FTIR_SVR_, P_FTIR_MME_) in coal ash.

| Sample No | P_XRF_ | P_FTIR_PLR_ | P_FTIR_PLSR_ | P_FTIR_RF_ | P_FTIR_SVR_ | P_FTIR_MME_ |
| --- | --- | --- | --- | --- | --- | --- |
| 1 | 0.282 | 0.187 | 0.078 | 0.860 | 0.760 | 0.375 |
| 2 | 0.404 | 0.287 | 0.287 | 0.860 | 0.783 | 0.478 |
| 3 | 0.533 | 0.340 | 0.124 | 0.862 | 0.958 | 0.442 |
| 4 | 0.826 | 0.795 | 0.473 | 0.926 | 1.597 | 0.731 |
| 5 | 1.345 | 1.183 | 0.744 | 2.880 | 2.720 | 1.602 |
| 6 | 1.887 | 1.965 | 1.701 | 3.999 | 3.461 | 2.555 |
| 7 | 0.671 | 0.541 | 0.446 | 0.798 | 0.659 | 0.595 |
| 8 | 0.989 | 0.993 | 0.749 | 0.810 | 0.795 | 0.851 |
| 9 | 1.324 | 1.239 | 0.879 | 0.881 | 1.260 | 1.000 |
| 10 | 2.004 | 1.381 | 1.296 | 1.863 | 2.218 | 1.513 |
| 11 | 3.313 | 3.094 | 3.241 | 2.964 | 2.797 | 3.100 |
| 12 | 4.697 | 4.545 | 5.000 | 3.442 | 3.652 | 4.329 |
| 13 | 0.588 | 0.504 | 0.593 | 0.809 | 0.450 | 0.635 |
| 14 | 0.862 | 0.972 | 1.140 | 0.809 | 0.578 | 0.974 |
| 15 | 1.148 | 1.238 | 1.520 | 0.857 | 0.816 | 1.205 |
| 16 | 1.756 | 1.842 | 2.623 | 1.762 | 1.655 | 2.076 |
| 17 | 2.860 | 3.147 | 3.953 | 2.505 | 2.411 | 3.201 |
| 18 | 4.047 | 4.452 | 6.053 | 3.427 | 3.332 | 4.644 |
| 19 | 0.428 | 0.639 | 0.129 | 0.829 | 0.816 | 0.532 |
| 20 | 0.617 | 0.727 | 1.197 | 0.831 | 0.858 | 0.918 |
| 21 | 0.822 | 0.935 | 1.392 | 0.845 | 1.092 | 1.057 |
| 22 | 1.230 | 1.414 | 2.407 | 1.780 | 2.090 | 1.867 |
| 23 | 2.111 | 2.920 | 2.671 | 3.155 | 3.043 | 2.915 |
| 24 | 2.929 | 3.281 | 3.205 | 4.304 | 3.865 | 3.597 |
| 25 | 0.783 | 0.896 | 0.945 | 0.794 | 0.650 | 0.878 |
| 26 | 1.219 | 0.758 | 0.841 | 0.794 | 0.801 | 0.798 |
| 27 | 1.546 | 1.658 | 1.683 | 0.794 | 0.870 | 1.378 |
| 28 | 2.447 | 2.284 | 2.405 | 1.329 | 1.447 | 2.006 |
| 29 | 3.989 | 4.024 | 4.236 | 1.860 | 2.426 | 3.374 |
| 30 | 5.596 | 5.324 | 5.782 | 3.080 | 3.708 | 4.729 |
| 31 | 0.392 | 0.405 | 0.111 | 0.841 | 0.756 | 0.452 |
| 32 | 0.545 | 0.492 | 0.049 | 0.849 | 0.809 | 0.464 |
| 33 | 0.723 | 0.755 | 0.313 | 0.939 | 1.213 | 0.669 |
| 34 | 1.144 | 1.193 | 0.740 | 1.849 | 1.919 | 1.261 |
| 35 | 1.901 | 1.417 | 0.926 | 3.242 | 3.006 | 1.862 |
| 36 | 2.604 | 2.011 | 2.266 | 4.805 | 4.228 | 3.027 |
| 37 | 0.728 | 1.055 | 0.746 | 0.796 | 0.811 | 0.865 |
| 38 | 1.075 | 1.433 | 1.066 | 0.830 | 0.938 | 1.110 |
| 39 | 1.374 | 1.568 | 1.328 | 1.562 | 1.705 | 1.486 |
| 40 | 2.064 | 2.500 | 2.583 | 2.767 | 3.187 | 2.617 |
| 41 | 3.470 | 3.699 | 3.327 | 3.687 | 4.256 | 3.571 |
| 42 | 4.916 | 4.128 | 3.197 | 4.613 | 4.777 | 3.979 |
| 43 | 0.575 | 0.735 | 0.745 | 0.722 | 0.750 | 0.734 |
| 44 | 0.862 | 0.661 | 0.841 | 1.135 | 0.865 | 0.879 |
| 45 | 1.122 | 0.762 | 0.872 | 0.998 | 0.932 | 0.877 |
| 46 | 1.718 | 1.502 | 2.270 | 2.312 | 2.446 | 2.028 |
| 47 | 2.853 | 2.762 | 3.158 | 2.821 | 3.374 | 2.914 |
| 48 | 4.027 | 3.633 | 3.764 | 4.708 | 4.482 | 4.035 |
| 49 | 0.792 | 0.703 | 0.167 | 0.780 | 0.306 | 0.550 |
| 50 | 1.101 | 0.993 | 0.675 | 0.786 | 0.775 | 0.818 |
| 51 | 1.494 | 1.306 | 0.957 | 0.904 | 1.075 | 1.056 |
| 52 | 2.229 | 1.437 | 0.982 | 1.405 | 1.155 | 1.275 |
| 53 | 3.760 | 2.554 | 2.121 | 2.948 | 2.369 | 2.541 |
| 54 | 5.326 | 3.691 | 3.986 | 4.610 | 4.280 | 4.096 |
| 55 | 0.673 | 0.757 | 0.698 | 0.803 | 0.642 | 0.753 |
| 56 | 0.980 | 0.780 | 0.655 | 0.808 | 0.779 | 0.748 |
| 57 | 1.301 | 1.226 | 1.081 | 1.503 | 1.311 | 1.270 |
| 58 | 2.014 | 1.753 | 1.435 | 1.751 | 1.667 | 1.646 |
| 59 | 3.211 | 2.767 | 2.901 | 3.023 | 2.885 | 2.897 |
| 60 | 4.513 | 3.093 | 3.898 | 4.360 | 4.865 | 3.784 |
| 61 | 0.851 | 0.974 | 0.668 | 0.783 | 0.687 | 0.809 |
| 62 | 1.284 | 1.595 | 0.812 | 0.793 | 0.765 | 1.066 |
| 63 | 1.739 | 2.011 | 1.322 | 1.372 | 1.357 | 1.568 |
| 64 | 2.583 | 2.919 | 1.664 | 1.621 | 1.558 | 2.068 |
| 65 | 4.398 | 4.895 | 2.846 | 2.836 | 2.703 | 3.526 |
| 66 | 6.101 | 4.975 | 3.882 | 3.961 | 3.887 | 4.273 |
| 67 | 0.282 | 0.422 | 0.349 | 0.826 | 0.717 | 0.533 |
| 68 | 0.404 | 0.989 | 1.164 | 0.839 | 0.995 | 0.997 |
| 69 | 0.545 | 0.882 | 0.969 | 1.370 | 1.392 | 1.074 |
| 70 | 0.831 | 1.483 | 1.778 | 1.787 | 1.761 | 1.683 |
| 71 | 1.339 | 1.839 | 2.155 | 3.111 | 2.913 | 2.368 |
| 72 | 1.879 | 2.367 | 3.192 | 4.265 | 3.918 | 3.275 |
| 73 | 0.801 | 0.684 | 0.417 | 0.786 | 0.363 | 0.629 |
| 74 | 1.211 | 0.965 | 0.790 | 0.792 | 0.754 | 0.849 |
| 75 | 1.582 | 1.128 | 1.024 | 1.074 | 1.104 | 1.076 |
| 76 | 2.325 | 1.597 | 1.699 | 1.724 | 1.714 | 1.673 |
| 77 | 3.905 | 2.055 | 2.355 | 2.731 | 2.344 | 2.380 |
| 78 | 5.509 | 3.662 | 4.608 | 4.447 | 4.020 | 4.239 |
| 79 | 0.501 | 0.164 | 0.327 | 0.816 | 0.864 | 0.436 |
| 80 | 0.718 | 0.345 | 0.390 | 0.825 | 1.212 | 0.520 |
| 81 | 0.945 | 0.497 | 0.449 | 0.850 | 1.395 | 0.599 |
| 82 | 1.410 | 0.935 | 0.575 | 1.678 | 1.997 | 1.063 |
| 83 | 2.387 | 1.016 | 0.420 | 2.589 | 3.023 | 1.341 |
| 84 | 3.338 | 1.898 | 1.353 | 3.216 | 3.955 | 2.156 |
| 85 | 0.410 | 0.819 | 0.706 | 0.822 | 0.963 | 0.783 |
| 86 | 0.587 | 1.259 | 1.218 | 1.226 | 1.396 | 1.234 |
| 87 | 0.783 | 1.270 | 1.157 | 1.307 | 1.353 | 1.245 |
| 88 | 1.180 | 1.471 | 1.271 | 1.754 | 1.641 | 1.499 |
| 89 | 1.979 | 2.586 | 2.675 | 3.090 | 3.035 | 2.784 |
| 90 | 2.756 | 4.341 | 5.474 | 5.112 | 5.886 | 4.976 |
| 91 | 0.931 | 0.669 | 0.837 | 0.803 | 0.829 | 0.770 |
| 92 | 1.417 | 0.966 | 1.522 | 1.152 | 1.262 | 1.213 |
| 93 | 1.840 | 1.332 | 1.723 | 1.625 | 1.505 | 1.560 |
| 94 | 2.724 | 2.075 | 2.476 | 2.008 | 2.136 | 2.186 |
| 95 | 4.663 | 2.587 | 2.985 | 2.701 | 2.428 | 2.758 |
| 96 | 6.510 | 3.667 | 4.592 | 4.367 | 4.011 | 4.208 |

(PLR: piecewise linear regression, PLSR: partial least square regression, RF: Random forest, SVR: Support vector regression)
