# Supplementary material for: A novel multi-model estimation of phosphorus in coal and its ash using FTIR spectroscopy
Source: Sci Rep. 2024 Jun 14;14:13785. doi: 10.1038/s41598-024-63672-x (PMC11637066; doi:10.1038/s41598-024-63672-x)
Supplement: Supplementary file 1 — Supplementary Information. [file 41598_2024_63672_MOESM1_ESM.zip › SI Guide.doc]

SI supplied as separate files: Supplementary Tables

The SI file consists of two tables labeled as Supplementary Table S1 and Supplementary Table S2. Supplementary Table S1 provides the comparison table of the phosphorus content of the 96 samples in parts per million (ppm) measured by XRF and determined by FTIR spectra-based machine learning techniques such as piece-wise linear regression (PLR), partial least square regression (PLSR), random forest (RF), support vector regression (SVR) and using the multi-model estimation (MME) approach in coal. Similarly, Supplementary Table S2 provides the comparison table of the phosphorus content of the 96 samples in parts per million (ppm) measured by XRF and determined by FTIR spectra-based machine learning techniques such as PLR, PLSR, RF, SVR, and MME techniques in coal ash.
